# Lower [^18^F]fallypride binding to dopamine D_2/3_ receptors in frontal brain areas in adults with 22q11.2 deletion syndrome: a positron emission tomography study

**DOI:** 10.1017/S003329171900062X

**Published:** 2020-04

**Authors:** Esther D. A. van Duin, Jenny Ceccarini, Jan Booij, Zuzana Kasanova, Claudia Vingerhoets, Jytte van Huijstee, Alexander Heinzel, Siamak Mohammadkhani-Shali, Oliver Winz, Felix Mottaghy, Inez Myin-Germeys, Thérèse van Amelsvoort

**Affiliations:** 1 Department of Psychiatry & Neuropsychology, Maastricht University, Maastricht, The Netherlands; 2 Department of Nuclear Medicine and Molecular Imaging, Division of Imaging and Pathology, University Hospital Leuven, KU Leuven, Belgium; 3 Academic Medical Center, Amsterdam, The Netherlands; 4 Department of Neuroscience, Center for Contextual Psychiatry, KU Leuven – Leuven University, Leuven, Belgium; 5 Department of Nuclear Medicine, University Hospital RWTH, Aachen University, Aachen, Germany; 6 Department of Radiology and Nuclear Medicine, Maastricht University Medical Center (MUMC+), Maastricht, The Netherland

**Keywords:** [18F]fallypride, 22q11DS, COMT, dopamine, frontal, PET

## Abstract

**Background:**

The 22q11.2 deletion syndrome (22q11DS) is caused by a deletion on chromosome 22 locus q11.2. This copy number variant results in haplo-insufficiency of the catechol-O-methyltransferase (COMT) gene, and is associated with a significant increase in the risk for developing cognitive impairments and psychosis. The COMT gene encodes an enzyme that primarily modulates clearance of dopamine (DA) from the synaptic cleft, especially in the prefrontal cortical areas. Consequently, extracellular DA levels may be increased in prefrontal brain areas in 22q11DS, which may underlie the well-documented susceptibility for cognitive impairments and psychosis in affected individuals. This study aims to examine DA D_2/3_ receptor binding in frontal brain regions in adults with 22q11DS, as a proxy of frontal DA levels.

**Methods:**

The study was performed in 14 non-psychotic, relatively high functioning adults with 22q11DS and 16 age- and gender-matched healthy controls (HCs), who underwent DA D_2/3_ receptor [^18^F]fallypride PET imaging. Frontal binding potential (BP_ND_) was used as the main outcome measure.

**Results:**

BP_ND_ was significantly lower in adults with 22q11DS compared with HCs in the prefrontal cortex and the anterior cingulate gyrus. After Bonferroni correction significance remained for the anterior cingulate gyrus. There were no between-group differences in BP_ND_ in the orbitofrontal cortex and anterior cingulate cortex.

**Conclusions:**

This study is the first to demonstrate lower frontal D_2/3_ receptor binding in adults with 22q11DS. It suggests that a 22q11.2 deletion affects frontal dopaminergic neurotransmission.

## Introduction

The 22q11.2 deletion syndrome (22q11DS) is a relatively common genetic disorder, with an estimated prevalence of one in 2000–4000 births. It is characterized by a deletion on locus 22q11.2, a copy number variant that contributes significantly to the risk for psychotic disorders (Murphy *et al*., 1999; Schneider *et al*., 2014). 22q11DS has a heterogeneous phenotype including cardiac anomalies (Guo *et al*., 2017) and several psychiatric problems (Schneider *et al*., 2014). Cognitive impairments (Oskarsdóttir *et al*., 2004; Bassett *et al*., 2005; Biswas and Furniss, 2016; Norkett *et al*., 2017) are part of the core symptoms of the syndrome. Additionally, approximately one in four individuals with 22q11DS develop a psychotic disorder, making 22q11DS one of the greatest known risk factors for developing psychosis (Bassett, 2011).Therefore, it is suggested that 22q11DS represents a valuable model for the study of neurobiological factors underlying both cognitive impairments (Oskarsdóttir *et al*., 2004; Bassett *et al*., 2005; Biswas and Furniss, 2016; Norkett *et al*., 2017) and psychotic disorders (Gur *et al*., 2017). Although the biological factors underlying psychotic disorders and (their) cognitive symptoms are still poorly understood, there is evidence suggesting for aberrant dopamine (DA) levels in several brain regions (Howes *et al*., 2012; Fusar-Poli and Meyer-Lindenberg, 2013), including the prefrontal cortex (PFC) (Slifstein *et al*., 2015).

Alterations in DA neurotransmission are also suggested to underlie some of the psychiatric problems typically seen in 22q11DS (Boot *et al*., 2008, 2011*a*; Evers *et al*., 2014; de Koning *et al*., 2015). These alterations are possibly due to haplo-insufficiency (reduced dosage of the gene due to hemizygosity) of the catechol-O-methyltransferase (COMT) gene, located on the deleted region and coding for the enzyme that catabolizes extracellular DA (Chen *et al*., 2004). Especially frontal DA is thought to be affected by COMT haploinsufficiency (Yavich *et al*., 2007) in 22q11DS. This could be explained by the relatively low density of the DA transporter in the PFC (Sesack *et al*., 1998), resulting in a DA dependency of COMT enzyme activity for clearance (Tunbridge *et al*., 2007). It has been indicated that 50% of the prefrontal DA clearance results from COMT activity (Yavich *et al*., 2007). Since patients with 22q11DS have only one copy of the COMT gene, which is associated with reduced COMT gene expression (van Beveren *et al*., 2012) and enzyme concentrations (Gothelf *et al*., 2014), they may consequently be chronically exposed to abnormally high DA levels (Boot *et al*., 2008), particularly in the PFC. We previously showed that the COMT functional polymorphism Val158Met indeed affects DA function in 22q11DS (Boot *et al*., 2011*b*). 22q11DS Val-hemizygotes have higher post-synaptic striatal DA D_2/3_ non-displaceable receptor binding potential (D_2/3_R BP_ND_) compared to carriers with the relatively unstable and less active COMT Met-allele (Boot *et al*., 2011*b*), further implicating altered DA neurotransmission.

The COMT Val/Met genotype has also been related to (dys)function of frontal brain regions in the psychosis continuum (Egan *et al*., 2001; Hernaus *et al*., 2013). Abnormalities in frontal brain DA have been hypothesized to especially underlie cognitive and negative symptoms of psychotic disorders (Howes and Kapur, 2009; Howes *et al*., 2012), which may also be true for 22q11DS (Stoddard *et al*., 2010; Schneider *et al*., 2014; Tang *et al*., 2014). Frontal DA neurotransmission has also been related to (impairments in) different neuropsychological functional domains, including memory, motivation, attention, and concentration (Howes and Kapur, 2009; Jonas *et al*., 2014; Slifstein *et al*., 2015). In addition, the COMT genotype is found to modulate cognitive functioning, relying on frontal DA neurotransmission, in psychotic disorder (Jonas *et al*., 2014; Slifstein *et al*., 2015) and in 22q11DS (Gothelf *et al*., 2005; de Koning *et al*., 2012; Carmel *et al*., 2014). Moreover the COMT genotype has been implicated in dopaminergic drug effects on cognitive functioning (Schacht, 2016).

In summary, there is evidence for abnormal frontal DA functioning in cognitive impairments, psychotic disorders, and implications for altered DA function in 22q11DS. More insight into the neurobiological factors associated with both psychotic disorder and cognitive deficits in 22q11DS can be gained, by investigating frontal DA function in 22q11DS using *in vivo* molecular imaging methods.

Neuroimaging techniques consistently showed both aberrant frontal brain anatomy and function as well as an effect of COMT Val/Met genotype in 22q11DS (van Amelsvoort *et al*., 2001, 2008; Gothelf *et al*., 2005; Zinkstok and van Amelsvoort, 2005; Kates *et al*., 2006; Howes *et al*., 2012; Shashi *et al*., 2012; van Beveren *et al*., 2012; Jonas *et al*., 2014).

In addition, molecular imaging techniques, including [^11^C]DTBZ- and [^18^F]fallypride positron emission tomography (PET) and [^123^I]IBZM single photon emission computed tomography (SPECT), have been used successfully in 22q11DS to investigate abnormalities in the striatal DA system (Boot *et al*., 2010; Butcher *et al*., 2017; van Duin *et al*., 2018). However, no studies to date have investigated frontal DA signaling in patients with 22q11DS. This can be measured *in vivo* with PET, using high-affinity radioligands such as the highly selective DA D_2/3_ receptor (D_2/3_R) radioligand [^18^F]fallypride, successfully used to probe frontal DA functioning (Lataster *et al*., 2011; Ceccarini *et al*., 2012; Hernaus *et al*., 2013; Nagano Saito *et al*., 2013).

The present study aimed to investigate, for the first time, frontal D_2/3_R BP_ND_ in 22q11DS using [^18^F]fallypride PET. Because of COMT haploinsufficiency in 22q11DS and previously described findings of SPECT and PET studies (Boot *et al*., 2010, 2011*b*; Butcher *et al*., 2017; van Duin *et al*., 2018), we expected reduced D_2/3_R BP_ND_ in frontal brain regions compared to healthy controls (HCs), as a proxy marker of chronically increased extracellular frontal DA levels.

## Materials and methods

### Participants

Fourteen non-psychotic adult individuals (eight females and six males, mean age = 34.6 years, s.d. = 9.7 years) with 22q11DS and no family history of psychotic disorder were included. They were compared to a previously published (Kasanova *et al*., 2017, 2018) sample of 18 HCs (12 females and six males, mean age = 38.1 years, s.d. = 15.6 years). Recruitment and exclusion criteria of HC have been described previously (Kasanova *et al*., 2017, 2018).

All participants were capable of giving written informed consent and did so after receiving full information on the study. Participants were treated in accordance with the Declaration of Helsinki. The study was approved by the Medical Ethical Committee of Maastricht University (The Netherlands) and the RWTH Aachen University ethics committee of Universitäts Klinikum (Germany). The PET protocol was additionally approved by the national authority for radiation protection in humans in Germany (Bundesamt für Strahlenschutz, BfS). Participants received coupons with a total value of €100 for participating in the PET study.

Exclusion criteria for 22q11DS participants were: (1) lifetime history of psychosis as determined by the Mini-International Neuropsychiatric Interview (M.I.N.I.) (Sheehan *et al*., 1998) and/or current or previous use of antipsychotic or stimulant medication, (2) contraindications for MRI and/or PET imaging, (3) pregnancy (verified on the day of the scan using a pregnancy test), (4) current drug use (verified on the day of the scan using a urine drug test).

Two HC participants were cigarette smokers. Given the well-known association between smoking (status) and DA function (Mansvelder and McGehee, 2000), they were asked to refrain from nicotine use on the day of the imaging session. One HC was excluded due to positioning difficulties during scanning. Another HC participant was excluded based on non-compliance with the study procedures. Two 22q11DS participants used the selective serotonin reuptake inhibitors escitalopram (10 mg) or paroxetine (20 mg). Since this may influence DA functioning (Tanda *et al*., 1994; Damsa *et al*., 2004) they were asked to refrain from taking their medication on the day of the imaging session. Other participants did not take any psychotropic medication. The final sample consisted of 16 HC and 14 22q11DS participants (Table 1).

**Table 1.** Demographics and binding potential (BP_ND_) per region of interest (ROI)^c^

Between groups	22q11DS (*n* = 14)		HC (*n* = 16)			
	Mean	s.d.	Mean	s.d.	Test-stat.	*p* value
Demographics						
Age	34.57	9.73	38.06	15.61	−0.74^a^	0.48
IQ	79.14	12.47	103.75	8.14	−6.486^a^	<0.01**
Male | female (*n*)	6|8		4|12		1.07^b^	0.30
Smoking (*n*)	0		2			
Medication free (*n*)	12^c^		16			
PANSS total score	33.21	3.42				
PANSS positive symptoms	7.14	0.53				
PANSS negative symptoms	8.14	1.66				
PANSS general psychopathology	17.93	2.06				
BP_ND_^18^F-fallypride	Mean	s.d.	Mean	s.d.	*F*-test stat.	*p* value
ROIs						
PFC	0.34	0.11	0.43	0.11	4.91	0.035
OFC	0.65	0.26	0.77	0.27	1.47	0.236
ACC	1.08	0.43	1.18	0.41	0.40	0.530
Ant cingulate gyrus	0.35	0.10	0.49	0.11	12.07	0.002**

HC, healthy controls; IQ, intelligence quotient; PANSS, positive and negative symptom scale: total score rage min 30–max 210, positive and negative symptom score range min 7–max 49, general psychopathology score range min 16–max 112; PFC, prefrontal cortex; OFC, orbito frontal cortex; ACC, anterior cingulate cortex.

**p < 0.01 and survived Bonferroni correction for multiple testing a = *t* test, b = χ^2^ test, c = 2 participants with 22q11DS used selective serotonin reuptake inhibitors (SSRIs) escitalopram (10 mg) and paroxetine (20 mg).

### Behavioral and physiological assessments

Full scale intelligence quotient (IQ) of the 22q11DS participants was determined using a shortened Dutch version of the Wechsler Adult Intelligence Scale – III (WAIS-III) (Wechsler, 1997) and was assessed on the day of scanning or in a separate session before or after the PET session (mean = 52.8 days, s.d. = 49.8 days). The shortened WAIS-III consists of four subtests: arithmetic and information (verbal IQ) digit-symbol-coding and block patterns (performance IQ) (Wechsler, 1997; Brooks and Weaver, 2005). In the HC group, total IQ was estimated using the Dutch Adult Reading Test (DART) (Schmand *et al*., 1991). Other assessments of the HC group were described previously (Kasanova *et al*., 2017, 2018).

To assess the presence and severity of psychotic symptoms, the Positive and Negative Syndrome Scale (PANSS) (Kay *et al*., 1987) for psychotic disorders was used.

### Image data collection

**Fig. 1.**
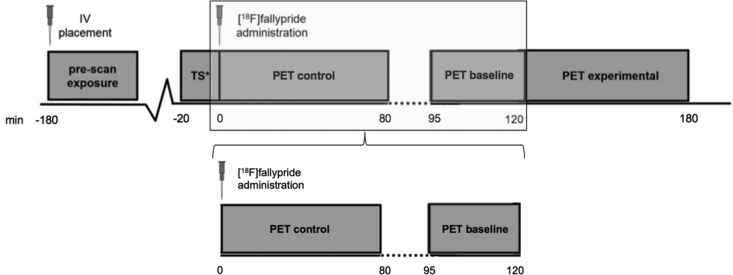
PET acquisition protocol. The original PET acquisition protocol. In gray, the part of the PET acquisition protocol used for analyses in this study is highlighted. *TS = ^68^Ge/^68^Ga-transmission scan, timeline in minutes. *PET control: Sensori-motor control condition*: Participants conducted a sensori-motor control condition prior to the baseline and experimental condition (previously described in Kasanova *et al*., 2017, 2018). This condition was designed to contain all features of the task of the experimental condition, without the main manipulation of the experimental condition; outcome-based associative learning. This control condition was presented on a 30-inch screen placed in the field of view of the participant. Similar to the experimental condition, images of a stimulus (photographs of actors) appeared on the screen and participants had to choose between one of two items depicted under the stimulus, for instance, indicate whether the actor was male or female, had short or long hair. The participant was instructed before the task that there was no right or wrong answer. No feedback was provided during the task. The control condition consisted of six blocks of 120 trials, in which 18 actors were presented 40 times, lasting approximately 10 min per block with intertrial intervals where the previous stimulus and items were still visible on the screen for 4 s. The sensori-motor control scan lasted 80 min and consisted of a total of 36 frames (6 × 60 s frames + 30 × 120 s frames). *PET baseline condition*: During the baseline condition the participants were instructed to lay down and rest in the scanner. The baseline scan lasted 25 min and consisted of 18 (120 s) frames.

The [^18^F]fallypride PET data collection acquired for this research was part of a comprehensive PET acquisition protocol, previously carried out to investigate reinforcement learning task-induced striatal DA release (Kasanova *et al*., 2017, 2018; van Duin *et al*., 2018). For the current PET analyses, only the [^18^F]fallypride sensorimotor control and baseline conditions were considered, including the first 120 min of the scan protocol (Fig. 1). All details of the whole PET procedure and the structural MRI and PET data acquisition have been described previously (Kasanova *et al*., 2017; van Duin *et al*., 2018) and additional analyses including the control only condition (excluding the 25 min baseline scan) to confirm reliability of the used method can be found in the Supplementary Materials.

### Image processing – dopamine D_2/3_ receptor binding potential maps – and analysis

**Fig. 2.**
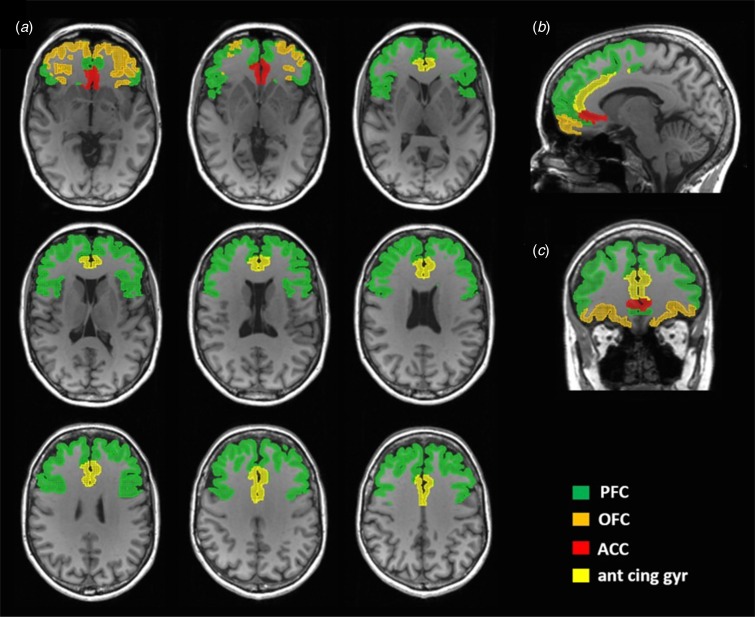
Masks for the frontal cortex. The mask is overlaid on a structural MRI scan and shown in transversal (*a*), sagittal (*b*), and coronal (*c*) views. MRI, magnetic resonance imaging; PFC, prefrontal cortex; OFC, orbitofrontal cortex; ACC, anterior cingulate cortex; ant cing gyr, anterior cingulate gyrus.

Image pre-processing procedures were performed as described previously (Kasanova *et al*., 2017, 2018; van Duin *et al*., 2018) using an automatic pipeline in the PMOD brain PNEURO tool (v. 3.8, PMOD Technologies, Zurich, Switzerland) (see Supplementary Materials). For each subject, individual voxel-wise parametric maps of DA D_2/3_R BP_ND_ (Innis *et al*., 2007) were generated in patient space using the Ichise's Multilinear Reference Tissue Model 2 (MRTM2) (Ichise *et al*., 2003). The cerebellum, including the cerebellar hemispheres without the vermis, was used as the reference region, because of its relative lack of D_2/3_R (Hall *et al*., 1994). The details of the MRTM2 analyses can be found in the Supplementary Materials. For the regional-based group comparison analysis (HC *v.* 22q11DS), a predefined prefrontal mask was generated in patient space for each subject according to the Hammers N30R83 atlas (Hammers *et al*., 2003). This predefined mask included composite and bilateral region of interests (ROIs), for: (1) PFC, including orbitofrontal cortex (OFC), inferior, middle, and superior frontal gyrus, (2) OFC only, including the anterior, medial, lateral, and parietal orbital gyrus, (3) anterior cingulate cortex (ACC), including only the subgenual and presubgenual ACC, and (4) anterior cingulate gyrus (Fig. 2 and online Supplementary Fig. S1).

### Statistical analyses

Statistical analyses were conducted in SPSS (IBM SPSS Statistics version 25.0). Between-group differences in demographic characteristics were investigated using χ^2^ and independent sample *t* tests. Average BP_ND_ values within each ROI (PFC, OFC, ACC, anterior cingulate gyrus) were determined and compared between the 22q11DS and HC group using analysis of variance. Post-hoc analyses were conducted to investigate group differences between HC and 22q11DS in BP_ND_ in all sub-regions of the frontal ROIs performing an analysis of variance. In the 22q11DS group, to investigate the relation between frontal BP_ND_, IQ, and PANSS scores, Pearson correlation coefficients were calculated with two-tailed tests of significance. The analyses were corrected for *n* = 4 ROIs, using a Bonferroni correction (critical *p* value *p* = 0.05/4 = 0.013).

## Results

### Demographic data

Sociodemographic variables of the sample are summarized in Table 1. There were no significant differences between the 22q11DS and the HC group in age (*t* = 0.74, *p* = 0.48) and gender distribution (22q11DS M/F ratio 6/8; HC M/F ratio 4/12; χ^2^ = 1.07, *p* = 0.30). As expected, IQ-scores were significantly lower in the non-psychotic [PANSS (Leucht *et al*., 2005) scores <58] 22q11DS group compared with the HC group (*t* = 6.48, *p* < 0.001), given that impaired cognitive functioning is a core characteristic of the syndrome (Jonas *et al*., 2014; Schneider *et al*., 2014; Weinberger *et al*., 2016).

### Frontal D_2/3_R BP_ND_ in 22q11DS *v*. HC

**Fig. 3.**
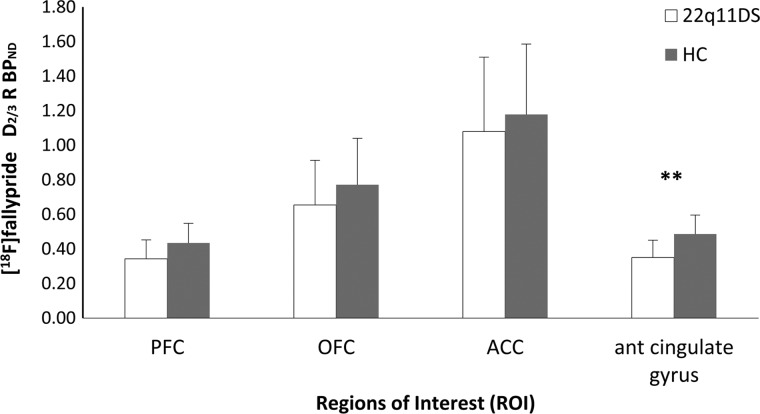
Binding potential (BP_ND_) per region of interest (ROI). Average dopamine D_2/3_ receptor binding potential (D_2/3_R BP_ND_) (*y*-axis) in the prefrontal cortex (PFC), the orbitofrontal cortex (OFC), the anterior cingulate cortex (ACC), and the anterior cingulate gyrus (*x*-axis). The healthy control (HC) group is depicted in gray and the 22q11DS group in white. Mean D_2/3_R BP_ND_ was significantly (**) lower in the 22q11DS group compared with the HC group in the anterior cingulate gyrus. Error bars represent standard deviation's (s.d.s). **p < 0.013 survived Bonferroni correction for multiple testing. HC, healthy controls.

Compared with HC, adults with 22q11DS revealed a significant lower D_2/3_R BP_ND_ in the PFC (*F* = 4.91, *p* = 0.035) and anterior cingulate gyrus (*F* = 12.07, *p* = 0.002) (see Table 1 and Fig. 3, individual data points are plotted in online Supplementary Fig. S2), suggesting lower receptor BP_ND_ in 22q11DS. There was no significant difference in D_2/3_R BP_ND_ between HC and adults with 22q11DS in the OFC and ACC (*F* = 1.47, *p* = 0.24 and *F* = 0.40, *p* = 0.53, respectively; Table 1 and Fig. 3). Results of separate sub-regions of the PFC, OFC, and ACC can be found in the online Supplementary Table S1 and Fig. S3. There was no significant association between D_2/3_R BP_ND_ in any of the frontal ROIs (*p* > 0.05) and IQ within the HC group and with IQ or PANSS scores within the 22q11DS group.

## Discussion

Here we report the results of the first study investigating frontal dopaminergic neurotransmission in 22q11DS, a genetic syndrome that is considered a valuable model for the study of biomarkers of psychotic disorders and cognitive deficits. As hypothesized, we found lower frontal D_2/3_ receptor BP_ND_ in adults with 22q11DS compared with HCs, indicating abnormal frontal DA levels in adults with 22q11DS.

### Lower frontal D_2/3_R BP_ND_ in 22q11DS

Lower D_2/3_R BP_ND_ in frontal brain regions adds to the growing evidence indicating aberrant DA neurotransmission in 22q11DS (Boot *et al*., 2008, 2010, 2011*a*; de Koning *et al*., 2012; Evers *et al*., 2014; Butcher *et al*., 2017; van Duin *et al*., 2018). There are several potential underlying mechanisms that could explain this novel finding.

It is thought that the radiotracer [^18^F]fallypride competes with endogenous DA levels for D_2/3_ receptor binding (Morris *et al*., 1995; Ceccarini *et al*., 2012). Lower receptor BP_ND_ can therefore be the result of a higher DA concentration in the synaptic cleft, which results in lower BP_ND_ due to competition and/or a down-regulation of post-synaptic DA receptor density (Wong *et al*., 1986; Boot *et al*., 2011*a*). This adds to accumulating evidence indicating a hyperdopaminergic state as a general endophenotype of 22q11DS in their young adulthood (Boot *et al*., 2008; Butcher *et al*., 2017). In line with current results, a recent PET study in non-psychotic adults with 22q11DS found higher pre-synaptic DA synthesis capacity in striatal brain regions (Butcher *et al*., 2017). A hyperdopaminergic state could be the result of reduced frontal DA clearance compared with HCs, caused by COMT haploinsufficiency in 22q11DS (Chen *et al*., 2004; Tunbridge *et al*., 2006). COMT hemizygosity in 22q11DS is suggested to result in reduced COMT enzyme activity and consequently higher DA levels, especially in the PFC (Tunbridge *et al*., 2006; Boot *et al*., 2008; van Beveren *et al*., 2012), in line with our findings. It has been suggested that the ‘clearance role’ of COMT and the effect of COMT Val/Met genotype in (frontal) DA turnover becomes increasingly important under challenged conditions (Huotari *et al*., 2002; Yavich *et al*., 2007), for instance during stress task-induced DA release paradigms (Hernaus *et al*., 2013). Future studies, possibly using a challenge condition and larger samples, are necessary to elaborate on the role of COMT genotype on frontal DA functioning in 22q11DS.

Furthermore, a chronic exposure to higher endogenous DA could have a toxic effect on dopaminergic neurons and is proposed to precede the onset of DA denervation in 22q11DS which is, amongst others, implicated in Parkinson's disease (PD) (Goldstein *et al*., 2014; Butcher *et al*., 2017). Recent studies indeed show that 22q11DS patients older than 30–40 years have an increased risk for the development of PD (Booij *et al*., 2010; Butcher *et al*., 2017), further linking abnormal dopaminergic neurotransmission to 22q11DS.

It is interesting to speculate about the clinical implications of the observed lower frontal D_2/3_ BP_ND_ and the proposed hyperdopaminergic state. On the one hand our results may be associated with cognitive impairments often seen in 22q11DS (Oskarsdóttir *et al*., 2004; Bassett *et al*., 2005; Biswas and Furniss, 2016; Norkett *et al*., 2017). Abnormal frontal DA levels may play a role in the induction of cognitive deficits based on the inverted U-shaped curve model (Goldman-Rakic *et al*., 2000; Gothelf *et al*., 2008). Thus the lower frontal D_2/3_ BP_ND_ in 22q11DS could be the result of excessive DA levels inducing cognitive deficits, including deficits in memory, attention, and reward processing (Gothelf *et al*., 2008). Such cognitive domains have previously been shown (using e.g. single-cell recordings and PET imaging) to rely, amongst others, on frontal DA functioning (Goldman-Rakic *et al*., 2000; Slifstein *et al*., 2015) and several of these cognitive domains have been found to be impaired in 22q11DS (de Koning *et al*., 2012; Weinberger *et al*., 2016; Norkett *et al*., 2017; van Duin *et al*., 2018). Future research including a comprehensive cognitive assessment tool is necessary, in order to associate cognitive functioning with frontal DA neurotransmission in 22q11DS.

Abnormal frontal DA levels could furthermore be related to the increased risk for developing psychotic disorders in 22q11DS. Problems in the cognitive domain often occur in psychotic disorders (Green and Nuechterlein, 1999; Nuechterlein *et al*., 2004).

Moreover, the severity of (primarily) cognitive and negative symptoms of psychotic disorders relying on frontal DA function (Okubo *et al*., 1997; Abi-Dargham *et al*., 2002; Slifstein *et al*., 2015) is likely to be associated with decreased DA release in frontal brain regions (Okubo *et al*., 1997). Although a frontal hypodopaminergic state is proposed to be related to non-deleted psychosis (Slifstein *et al*., 2015), we found lower frontal D_2/3_R BP_ND_ suggestive of a frontal hyperdopaminergic state and/or lower expression of post-synaptic DA receptor density (Wong *et al*., 1986; Boot *et al*., 2011*a*, 2011*b*) in non-psychotic adults with 22q11DS with (mild) cognitive impairments. This might be explained by the same mechanism as is proposed to result in cognitive dysfunction with the inverted U-shaped curve model (Goldman-Rakic *et al*., 2000). This model suggests that either too much or too little frontal DA levels induce cognitive deficits, which could also be true for psychosis-related symptoms. It could additionally be explained by previously found differences in DAergic markers in 22q11DS compared with individuals with ultra-high risk (Vingerhoets *et al*., 2018). Disturbances of the DAergic system in the pathway to psychosis may be different in the 22q11DS population compared with other risk groups.

However, direct evidence for frontal dopaminergic alterations in psychotic disorders is inconsistent and previous findings are inconclusive (Kambeitz *et al*., 2014). In this study, we found results indicating a hyperdopaminergic state in non-psychotic 22q11DS individuals, suggesting that frontal dopaminergic alterations are present in this group regardless of psychopathology. Future research in a sample including also patients with psychotic symptoms with 22q11DS would be interesting to provide additional insight in the association between psychotic risk and frontal DA functioning.

### Strengths and limitations

The main strength of this study is the use of a unique patient group with a well-defined genetic syndrome which is a valuable model for the study of biomarkers underlying, among others, cognitive impairments and psychotic disorders. Some limitations of the study should also be taken into account. First, the relatively small sample size of the sample and the use of antidepressant medication in some of the participants. We reanalyzed our main analyses excluding the 22q11DS subjects with medication and replicated our findings, indicating that the results were not affected by medication. Given the challenge of recruitment of (medication-naive) participants, the 22q11DS sample (size) could be considered representative, also in light of previous studies using similar paradigms (Hernaus *et al*., 2013; Kasanova *et al*., 2017; van Duin *et al*., 2018).

Secondly, given the well-known association between smoking (status) and DA function (Mansvelder and McGehee, 2000), we reanalyzed our main analyses excluding the HC subjects that were habitual cigarette smokers and replicated our findings, indicating that the results were not affected by smoking status.

Additionally, the design of the scanning protocol may also have affected the results, and should be taken into consideration in future research. For the analysis of ‘relative resting state’ DA levels, from the original protocol, the sensorimotor control and baseline condition were analyzed, without the experimental condition (designed to induce reward-related DA release) (Kasanova *et al*., 2017, 2018; van Duin *et al*., 2018). This design is necessary to detect reliable task-induced changes on the [^18^F]fallypride uptake (Vernaleken *et al*., 2011). A sensorimotor control task was used to control for sensorimotor influence on the experimental reward task condition and to keep subjects awake, in order to prevent unpredictable head movement. Although the subjects were well instructed before the sensorimotor control task (Fig. 1), the task might have influenced and elicited (sensorimotor-induced) DA release in frontal brain regions. However, this would have been the case for both the control and the 22q11DS group, and there is no evidence, to the best of our knowledge, to suggest that 22q11DS confers a different DA release to sensorimotor tasks compared with controls.

Furthermore, lower D_2/3_R BP_ND_ was found in the PFC and the anterior cingulate gyrus, however only the difference in the anterior cingulate gyrus survived the Bonferroni correction. Although D_2/3_R BP_ND_ seemed also lower in the OFC and ACC in 22q11DS compared with controls, this difference failed to reach significance. This could be due to a power issue and in increased sample sizes it is expected to find significant differences in these regions as well. More research is necessary to further explain the absence of significant differences in the OFC and ACC.

## Conclusion

This study is the first to demonstrate lower frontal dopamine D_2/3_ receptor binding in adults with 22q11DS, which may represent a hyperdopaminergic state in frontal brain areas. This could be the result of haplo-insufficiency of COMT in these patients, and may play a role in their increased risk for developing cognitive impairments and psychotic disorders.

## Supplementary material

For supplementary material accompanying this paper visit http://dx.doi.org/10.1017/S003329171900062X. click here to view supplementary material
