# Supplementary material for: Lower [18F]fallypride binding to dopamine D2/3 receptors in frontal brain areas in adults with 22q11.2 deletion syndrome: a positron emission tomography study
Source: Psychol Med. 2019 Apr 2;50(5):799–807. doi: 10.1017/S003329171900062X (PMC7168654; doi:10.1017/S003329171900062X)

**Supplementary Material**

*Structural MRI data acquisition*

First whole brain T1-weighted MRIs were collected before the PET scans were obtained. As part of other studies previously conducted (van Duin *et al.* 2017; Kasanova *et al*. 2017), whole brain high-resolution T1-weighted MRIs were collected on 3 different machines. In the case of 4 participants (only 22q11DS), acquisition was performed in previous research, using a Philips 3 Tesla Intera MRI system equipped with a 6-channel sense head coil (scan parameters: repetition time (TR) = 9.8 ms, echo time (TE) = 4.6 ms; matrix size = 192 × 152; slice thickness = 1.2 mm; 120 slices). For 9 participants (only 22q11DS), a high-resolution T1-weighted MRI scan was acquired (as part of another study) on a Siemens 7 Tesla Magnetom whole body MR system equipped with a 32-channel head coil. T1-weighted images were acquired using a MP2RAGE sequence (TR = 4500 ms; TE = 2.39 ms; matrix size = 256 x 256; slice thickness = 0.9 mm; 192 slices; generalized autocalibrating partially parallel acquisitions (GRAPPA) = 3). Finally, for 17 participants (n=16 controls, and n=1 22q11DS participant), a Siemens 3 Tesla scanner (Siemens Healthcare, Munich, Germany) was used, using the Magnetization Prepared Rapid Acquisition Gradient-Echo (MP-RAGE) sequence (TR = 1900 ms; TE = 2.52 ms; matrix dimensions = 256 x 256; slice thickness = 1 mm; 176 slices).

*PET data acquisition*

At least 90 minutes before the start of the PET scan, a non-magnetic intravenous cannula was placed in the antecubital vein of the participant’s arm for the injection of the radiotracer (Figure 1). To minimize head movement, participants were positioned on the scanner bed with their head fixated using a firm strap. Before the start of the PET acquisition protocol, a 10-minute low dose 68Ge/68Ga transmission scan was obtained, followed by the different PET paradigm conditions (Figure 1). In line with other comparable studies (Lataster *et al.* 2011; Hernaus *et al.* 2013), a single bolus infusion PET paradigm was utilized, using the high-affinity and selective DA D_2/3_R radiotracer [^18^F]fallypride. More details on the modification of the [^18^F]fallypride radiosynthesis method are described previously (Lataster *et al.* 2011). Using a slow intravenous bolus administration, participants received [^18^F]fallypride (mean injected dose = 202.3 MBq, SD = 6.88 MBq; specific radioactivity > 3.7 GBq/µmol; radiochemical purity > 99.7%). The injection of the radiotracer was immediately followed by the collection the dynamic emission scans in three-dimensional mode, using a Siemens ECAT EXACT HR+ scanner (Siemens-CTY, Knoxville, TN, USA).

The entire original PET acquisition protocol lasted 180 minutes in order to be able to obtain reliable estimates for both striatal and extrastriatal reward-induced DA release (Duin *et al.* 2017; Kasanova *et al.* 2017b). Based on the aim of the study, the frames of the experimental condition were not used, causing the final protocol to consist of 120 minutes (Figure 1). [^18^F]fallypride steady-state conditions are attained sooner in frontal compared to striatal regions, because of the lower density of D_2/3_ receptors in the frontal than striatal brain areas, accounting for accurate frontal D_2/3_ BP_ND_ estimates in a 2 hour scanning protocol (Christian *et al.* 2000; Vernaleken *et al.* 2011; Ceccarini *et al.* 2012) in contrast to a protocol longer than 2 hours for striatal brain areas (Ceccarini *et al.* 2012).

First, an 80-minute sensory-motor control condition was used, consisting of a total of 36 frames (6 x 60-second frames + 30 x 120-second frames). Then participants were removed from the scanner bed for a 15-minute break. They were repositioned using the localization system of the scanner and a 25-minute baseline rest images were obtained, consisting of 18 frames (120-second as frame length).

A dynamic frame was collected every 60 seconds during the first 6 minutes of the protocol. During the following 114 minutes (the remainder of the protocol), every 120 seconds PET data were collected, with a total of 63 frames, including the frames when the participant was outside of the scanner during the break. Data sets (slice thickness = 2.425 mm; pixel size = 2 x 2 mm) were reconstructed by filtered back projection (Hamm filter) after Fourier rebinning into two-dimensional sonograms, corrected for random coincidences, scatter and attenuation using the 10-minute ^68^Ge/^68^Ga transmission scan.

*PET data analyses*

Multi-frames [^18^F]fallypride PET images were first realigned with the average image of the complete 120 minute acquisition for motion correction. The dynamic motion-corrected [^18^F]fallypride PET image was then rigidly coregistered to the corresponding individual volumetric T1-weighted MR images, obtaining [^18^F]fallypride data in subject native space. The individual T1-weighted MR images were then nonlinearly coregistered to the standard Montreal Neurological Institute (MNI) space MRI template. Subsequently the same was done for the PET images using the same spatial transformation as the registered MR images. T1-weighted MR images were segmented into grey matter (GM), white matter and cerebrospinal fluid within native MRI space to automatically generate a total of 83 individual regions of interest (ROIs) according to the Hammers N30R83 atlas (Hammers *et al.* 2003). Automatic delineation of the deep nuclei, was performed by T1-weighted MRI parcellation in the PMOD PNEURO tool.

For each subject, individual voxel-wise parametric maps of DA D_2/3_ BP_ND_ (Innis *et al.* 2007) were generated in patient space using the Ichise's Multilinear Reference Tissue Model 2 (MRTM2)(Ichise *et al.* 2003). The MRTM2 is an adapted version of Ichise´s initial multilinear reference tissue model (MRTM) reducing the numbers of parameters to two by fixing the efflux rate constant of the ligand from the reference region (k_2_´) in all regions to the individual k_2_´ value gained from a preceding MRTM analysis of regions with low noise (i.e. high BP_ND_). In this way voxel-wise parameter estimation is less prone to bias due to the noise in the data. As suggested by Ichise and co-workers (Ichise *et al.* 2003), we determined a priori k_2_´ as the average of k_2_´ determined with MRTM in side-averaged putamen and caudate nucleus, regions with high BP_ND_. This k_2_´ values was then used for the voxelwise MRTM2 analysis.

The SRTM method is used over arterial input function method because the collecting of arterial blood samples adds risk, cost, measurement error, and patient discomfort to PET studies. Reference tissue methods have been found suitable for mapping striatal and extrastriatal regions with ^18^F-fallypride. The MRTM2 model, that was used in the current study to get BP_ND_ has been shown to be least sensitive to noise in the dynamic PET data, employing the use of a tissue reference region to represent the kinetics of unbound radioligand in the tissue (Cunningham et al., 1991; Logan et al., 1996).

**References**

**Ceccarini J, Vrieze E, Koole M, Muylle T, Bormans G, Claes S, Van Laere K** (2012). Optimized in vivo detection of dopamine release using 18F-fallypride PET. Society of Nuclear Medicine *Journal of nuclear medicine : official publication, Society of Nuclear Medicine* **53**, 1565–72.

**Christian B, Narayanan T, Shi B, Mukherjee J** (2000). Quantitation of striatal and extrastriatal D-2 dopamine receptors using PET imaging of [F-18]fallypride in nonhuman primates. *Synapse* **38**, 71–79.

**Duin EDA Van, Kasanova Z, Ceccarini J, Hernaus D, Heinzel A, Mottaghy F, Booij J, Myin-germeys I, van Amelvoort T** (2017). Reward Learning and Dopamine Release in Adults with 22q11DS: A Study Using [18F]fallypride Positron Emission Tomography. . Elsevier *Biological Psychiatry - Conference Abstract SOBP* **81**, S253–S254.

**Hammers A, Allom R, Koepp MJ, Free SL, Myers R, Lemieux L, Mitchell TN, Brooks DJ, Duncan JS** (2003). Three-dimensional maximum probability atlas of the human brain, with particular reference to the temporal lobe. . Wiley Subscription Services, Inc., A Wiley Company *Human Brain Mapping* **19**, 224–247.

**Hernaus D, Collip D, Lataster J, Ceccarini J, Kenis G, Booij L, Pruessner J, van Laere K, van Winkel R, van Os J, Myin-Germeys I** (2013). COMT Val158Met Genotype Selectively Alters Prefrontal [18F]Fallypride Displacement and Subjective Feelings of Stress in Response to a Psychosocial Stress Challenge. *PLoS ONE* **8**

**Ichise M, Liow J-S, Lu J-Q, Takano A, Model K, Toyama H, Suhara T, Suzuki K, Innis RB, Carson RE** (2003). Linearized Reference Tissue Parametric Imaging Methods: Application to [ ^11^ C]DASB Positron Emission Tomography Studies of the Serotonin Transporter in Human Brain. *Journal of Cerebral Blood Flow & Metabolism* **23**, 1096–1112.

**Innis RB, Cunningham VJ, Delforge J, Fujita M, Gjedde A, Gunn RN, Holden J, Houle S, Huang S-C, Ichise M, Iida H, Ito H, Kimura Y, Koeppe RA, Knudsen GM, Knuuti J, Lammertsma AA, Laruelle M, Logan J, Maguire RP, Mintun MA, Morris ED, Parsey R, Price JC, Slifstein M, Sossi V, Suhara T, Votaw JR, Wong DF, Carson RE** (2007). Consensus Nomenclature for *in vivo* Imaging of Reversibly Binding Radioligands. *Journal of Cerebral Blood Flow & Metabolism* **27**, 1533–1539.

**Kasanova Z, Ceccarini J, Frank MJ, Amelsvoort T van, Booij J, Heinzel A, Mottaghy F, Myin-Germeys I** (2017b). Striatal dopaminergic modulation of reinforcement learning predicts reward—oriented behavior in daily life. *Biological Psychology* **127**, 1–9.

**Lataster J, Collip D, Ceccarini J, Haas D, Booij L, Van Os J, Pruessner J, Laere K Van, Myin-Germeys I** (2011). Psychosocial stress is associated with in vivo dopamine release in human ventromedial prefrontal cortex: A positron emission tomography study using [ 18 F]fallypride. *NeuroImage* **58**, 1081–1089.

**Vernaleken I, Peters L, Raptis M, Lin R, Buchholz H-G, Zhou Y, Winz O, Rösch F, Bartenstein P, Wong DF, Scháfer WM, Gründer G** (2011). The applicability of SRTM in [ ^18^ F]fallypride PET investigations: Impact of scan durations. *Journal of Cerebral Blood Flow & Metabolism* **31**, 1958–1966.

**Supplementary Figure 1:** Masks for the sub-frontal regions.


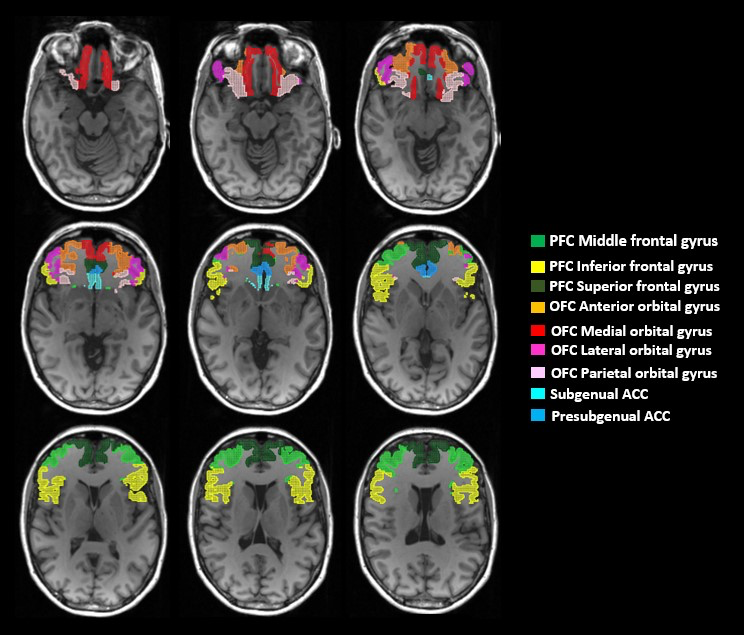


The masks are overlaid on a structural MRI scan and shown in transversal view.

MRI = magnetic resonance imaging; PFC = prefrontal cortex; OFC = orbitofrontal cortex; ACC = anterior cingulate cortex.

**Supplementary figure 2. D_2/3_R BP_ND_ in 22q11DS and controls plotted.**

**
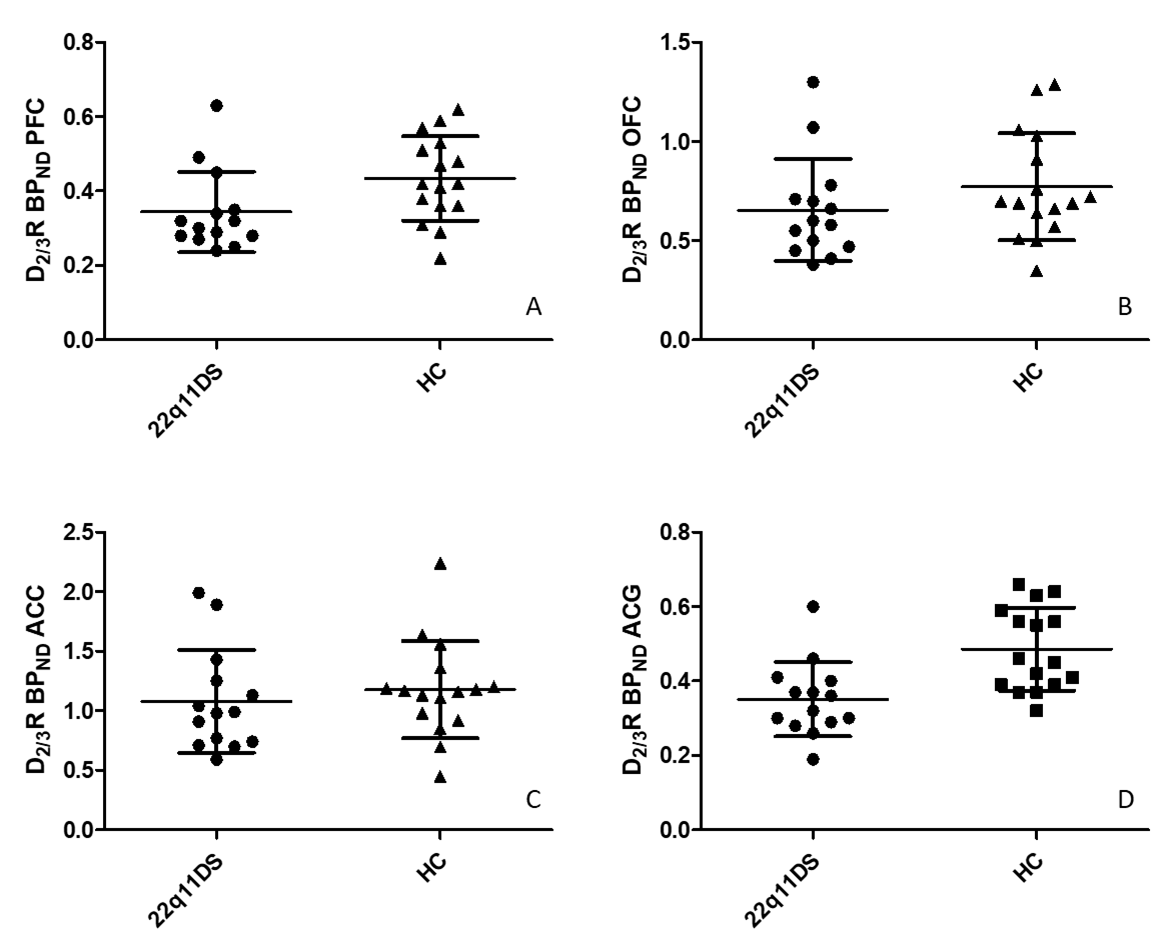
**

Patients with 22q11DS show *non* significant lower D_2/3_R BP_ND_ in the A) Prefrontal cortex (PFC), B) orbital frontal cortex (OFC), C) Anterrior cingulate cortex (ACC) compared to healthy controls (HC). D_2/3_R BP_ND_ was significantly lower in D) the anterior cingulate gyrus (ACG) in 22q11DS compared to controls following Bonferroni correction.

**Supplementary Table 1:** Binding potential (BP_ND_) per sub-region of interest (ROI)

| ***Between groups*** | ***22q11DS (n=14)*** | | ***HC (n=16)*** | |  |  |
| --- | --- | --- | --- | --- | --- | --- |
|  | **Mean** | **SD** | **Mean** | **SD** | **Test-stat.** | **p-value** |
| ***BP_ND_ ^18^F-fallypride*** |  |  |  |  |  |  |
| ***Subregions*** |  |  |  |  |  |  |
| ***PFC*** |  |  |  |  |  |  |
| *PFC Middle frontal gyrus* | 0.30 | 0.09 | 0.37 | 0.11 | 4.53^c^ | 0.042 |
| *PFC inferior frontal gyrus* | 0.31 | 0.10 | 0.39 | 0.10 | 5.02^c^ | 0.033 |
| *PFC superior frontal gyrus* | 0.25 | 0.09 | 0.34 | 0.10 | 6.48^c^ | 0.017 |
| ***OFC*** |  |  |  |  |  |  |
| *OFC* Anterior orbital gyrus | 0.24 | 0.09 | 0.38 | 0.13 | 9.83^c^ | 0.004^**^ |
| *OFC* Medial orbital gyrus | 0.85 | 0.40 | 1.00 | 0.43 | 0.98^c^ | 0.332 |
| *OFC* Lateral orbital gyrus | 0.34 | 0.08 | 0.42 | 0.14 | 3.91^c^ | 0.058 |
| *OFC* Parietal orbital gyrus | 1.15 | 0.47 | 1.20 | 0.48 | 0.09^c^ | 0.764 |
| ***ACC*** |  |  |  |  |  |  |
| *Subgenual ACC* | 1.70 | 0.73 | 1.81 | 0.77 | 0.16^c^ | 0.692 |
| *Presubgenual ACC* | 0.40 | 0.19 | 0.52 | 0.14 | .4.00.^c^ | 0.055 |

**p<0.006 survived Bonferroni correction for multiple testing in 9 ROIs (p=0.05/9=0.006) c=F-test; HC=healthy controls; PFC=prefrontal cortex; OFC=orbitofrontal cortex; ACC=anterior cingulate cortex.

**Supplementary Figure 3:** Binding potential (BP_ND_) per sub-region of interest (ROI)


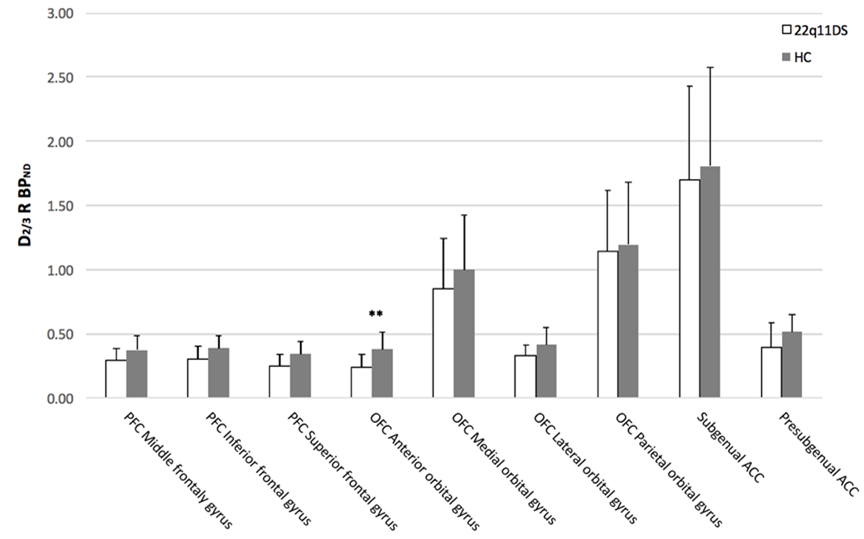


Average dopamine D_2/3_ receptor binding potential (D_2/3_R BP_ND_) (y-axis) in the subregions of the prefrontal cortex (PFC), the orbitofrontal cortex (OFC) and the anterior cingulate cortex (ACC) (x-axis). The healthy control (HC) group is depicted in grey and the 22q11DS group in white. After Bonferroni correction, mean D_2/3_R BP_ND_ was significantly (**) lower in the 22q11DS group compared to the HC group in one subregion of the OFC (anterior orbital gyrus).

Error bars represent standard deviation’s (SD’s). **p<0.006 survived Bonferroni correction for multiple testing HC= healthy controls.

**Supplementary Table 2:**

|  | **Control condition** | **Control + baseline condition** |
| --- | --- | --- |
| **MRTM - K_2_´** |  |  |
| Caudate nucleus | 0.253 | 0.210 |
| Putamen | 0.275 | 0.235 |
| **MRTM - BP_ND_** |  |  |
| Caudate nucleus | 15.52 | 19.05 |
| Putamen | 16.94 | 19.93 |
|  |  |  |
| MRTM2 - K_2_´- Putamen | 0.264 | 0.223 |
| MRTM2 - BP_ND_ - Putamen | 17.21 | 20.12 |
|  |  |  |

The ^18^F-fallypride kinetic behavior shows a marked effect of D2/3 receptor concentration on the equilibrium time of the radioligand and hence on the corresponding BP_ND_ estimation *[Vernaleken J Cereb Blood Flow Metabolism 2011]*. It takes long to reach equilibrium in the D2/3 receptor–rich regions (i.e. striatum) and therefore to obtain a reliable estimate for BP_ND_ in striatal regions (needed for MRTM / MRTM2). Therefore, applying the MRTM (or MRTM2) on the first part of the baseline scan only (control condition) covering only 80 minutes might not ensure parameter stability and reliability. This is described by a substantial change in BP_ND_ estimates when the baseline condition is excluded.

Indeed, when we have first performed the MRTM to estimate K_2_´ on the striatal regions including only the control condition, we have obtained higher values for the efflux rates from the reference region (K_2_´), and lower BP_ND_ (~20%) compared to the control + baseline condition. This striatal BP underestimation was reflected in the lower prefrontal BP values obtained when only the control condition was included (in average -11 ± 4 %), compared to the current used control and baseline condition, demonstrating the fact that 80 mins are not sufficient to properly evaluate ^18^F-fallypride BP_ND_. (See please the values reported in supp. Table 2 and figure 4 corresponding to one representative subject).

**Supplementary Figure 4:**


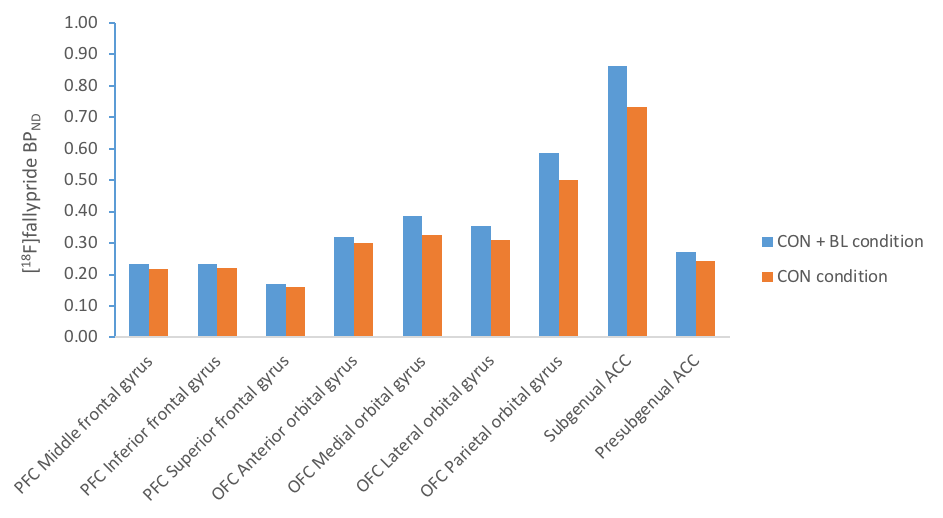

Supplement: Supplementary file 1 [file S003329171900062Xsup001.docx]
